# Gross Hematuria and Hemolytic Anemia in Infectious Mononucleosis

**DOI:** 10.1155/2024/5236969

**Published:** 2024-09-12

**Authors:** Chinmayi Sharma, Navneet Venugopal, Shivaiah Balachandra

**Affiliations:** ^1^ AJ Institute of Medical Sciences and Research Center, Mangaluru, India; ^2^ Department of Pediatrics The University of Texas Medical Branch, Galvestion, TX, USA; ^3^ Division of Pediatric Nephrology The University of Texas Medical Branch, Galveston, TX, USA

## Abstract

*Introduction*. Infectious mononucleosis (IM), caused by the Epstein–Barr virus (EBV), typically presents with fever, tonsillopharyngitis, and lymphadenopathy while rare, renal, and hematological complications such as gross hematuria and hemolytic anemia can occur, particularly in children. *Case Presentation*. We describe a 15-year-old male with infectious mononucleosis presenting with abdominal pain, sore throat, and red-colored urine for three days. Laboratory findings revealed leukocytosis, elevated liver enzymes, and hemoglobinuria. Serological testing confirmed EBV infection. Despite intravascular hemolysis, the patient's anemia was mild, and he responded well to supportive care. *Discussion*. Gross hematuria and hemolytic anemia are uncommon in IM, with limited reports. Proposed mechanisms include viral-induced renal injury and autoimmune hemolysis. Differential diagnosis should consider other causes of hematuria, which can be differentiated through urine microscopy and serological tests. IM should be considered in patients with pharyngitis and hematuria, especially when other causes are excluded.

## 1. Introduction

Infectious mononucleosis (IM), caused by the Epstein–Barr virus (EBV) of the herpes family, represents a spectrum of diseases that are typically characterized by fever, tonsillopharyngitis, hepatosplenomegaly, lymphadenopathy, and fatigue. Many multiorgan system complications have been reported, including CNS, hematological, and cardiac manifestations. Renal complications, specifically microscopic hematuria, have been reported with an incidence of up to 15%, and gross hematuria is a rare presenting symptom, with only a handful of case reports in the literature describing this association with EBV infection in children [1–5]. Hemolytic anemia is another uncommon complication associated with acute EBV infection with a reported incidence of <3% [6]. Although usually mild and self-limited in presentation, the recognition of hemolysis and assessment of the degree of anemia is a critical part of the evaluation of the patient. This case report describes the case of a 15-year-old Caucasian male who presented with gross hematuria and hemoglobinuria and was diagnosed with infectious mononucleosis.

## 2. Case Presentation

A previously healthy 15-year-old Caucasian male was admitted to the pediatric inpatient floor with a 2-week history of abdominal pain, sore throat, fatigue, and red-colored urine for three days. Abdominal pain occurred in the left upper quadrant, occasionally radiating to the groin, and was described as “cramping” and “sharp” in nature. His urine remained red throughout the urinary stream, and there was no history of clot passage.

The patient also had a history of difficulty swallowing for approximately six days and felt like he had some lumps on the left side of his throat. The patient denied fever, rash, cough, sore throat, rhinorrhea, vomiting, or diarrhea.

On physical examination, the patient appeared afebrile with stable vital signs. The pharynx was erythematous, with enlarged tonsils bilaterally, and exudates were present on the left side. Neck examination revealed enlarged, tender, anterior, and posterior cervical lymph nodes. Abdominal examination revealed no organomegaly, and the remaining systemic examination results were unremarkable.

Laboratory studies showed a white blood cell count of 20,400, a hemoglobin count of 15.4 g/dl, and a platelet count of 162,000. The differential count was 22% segmented neutrophils, 26% lymphocytes, 25% atypical lymphocytes, and 16% bands. Serum electrolytes were normal, with a blood urea nitrogen (BUN) level of 13 mg/dl (normal, 7–20 mg/dl) and creatinine of 0.66 mg/dl (normal, 0.3–0.7 mg/dl). Liver enzymes were elevated, with alanine aminotransferase (ALT) of 232 *μ*/l, aspartate aminotransferase (AST) of 152 *μ*/l, and lactate dehydrogenase (LDH) levels of 232 *μ*/L, 152 *μ*/L, and 1,505 *μ*/l (normal, 140–280 *μ*/l). Urinalysis revealed brown, turbid urine with 3+ gross blood cells, >182 red blood cells/hpf, and 1 white blood cell/hpf. A spun urine sample showed a red-colored supernatant and cellular sediment at the bottom, which was visualized under a microscope. It showed numerous nondysmorphic red blood cells with no cellular casts. Urine culture results were negative and coagulation studies were within normal limits.

Serological testing for infectious mononucleosis was performed, and the diagnosis was confirmed. The rapid mono screen test was positive, as was the EBV IgM test, whereas the EBV IgG test was negative and indicative of active EBV infection. The patient underwent a detailed investigation for hematuria, including serum complements, and all tests were negative.

Since the urinary supernatant was red, it was sent for free hemoglobin testing and was strongly positive at 3+. Urine myoglobin was weakly positive at 1+ level. The plasma hemoglobin level was significantly elevated at 60 mg/dl (normal 0.5–5.0). The serum myoglobin level was normal at 15 ng/ml (normal, <121 ng/ml). The patient also had an elevated LDH level of 1505 *μ*/l. In addition to hematuria, the patient had hemoglobinuria, but there was no proteinuria or glucosuria. Additional investigations for intravascular hemolysis could not be performed because the patient was doing well and requested for discharge.

The patient received supportive care during his hospital stay and was discharged with a pediatric nephrology appointment four days later. At the follow-up appointment, repeat laboratory studies revealed a normal CBC with rare, atypical lymphocytes and stable BUN and creatinine levels. The patient reported continued hematuria, urinalysis in the clinic was significant for large amounts of blood, and microscopic visualization of the sample was unchanged from hospital admission. The patient did not return for follow-up visits and was contacted by phone two weeks later. The patient reported that he was doing well, and his urine had completely cleared.

## 3. Discussion

The first case report describing gross hematuria as a manifestation of infectious mononucleosis was published by Tidy and Morley in 1921. Of 270 cases studied, one exhibited gross hematuria, while 6% had microscopic hematuria [7]. Including Taub's 1966 case report, only 10 more cases of gross hematuria with infectious mononucleosis have been reported in the last 40 years. A case of gross hematuria was noted in one of 113 children in a report in 1985, although that patient had severe thrombocytopenia resulting in hematuria [8]. In Lewis' 1988 case report, gross hematuria was noted in a patient three weeks following the acute phase of infection [1]. Renal involvement in infectious mononucleosis is being increasingly reported in the literature. Our case report represents one of the only reports of infectious mononucleosis with gross hematuria on presentation (not associated with hematologic abnormalities) reported over the last four decades.

Gross hematuria associated with infectious mononucleosis is not associated with acute glomerulonephritis complications. Our patient showed no hypertension, elevated blood urea nitrogen, or creatinine levels. This contrasts with glomerulonephritis, which presents with high blood pressure and hematuria, and has a different prognosis. Gross hematuria generally resolves quickly without lasting renal damage [2].

The pathophysiology of gross hematuria in infectious mononucleosis is largely unknown. Older studies with postmortem renal biopsies of children who died from mononucleosis suggested renal tubular necrosis and lymphocytic aggregates in the renal cortex as possible mechanisms [3]. A 1998 study indicated that EBV, as a nephritogenic antigen, plays a critical role in renal injury in membranous nephropathy, IgA nephropathy, and focal/segmental lesions [9].

Our patient presented with gross hematuria and significant hemoglobinuria, indicating hemolysis. A 1973 Mayo Clinic study of 1380 patients with presumed infectious mononucleosis found only five with hemolytic anemia [10]. The literature shows that hemolytic signs typically develop one to two weeks after acute EBV infection, with anemia ranging from mild to severe [6]. Despite intravascular hemolysis in this case, the patient did not develop significant anemia.

Although several mechanisms have been proposed to explain the hemolytic process secondary to mononucleosis, including hypersplenism and direct action of the virus on erythrocytes, the presence of autoimmune hemolytic antibodies appears to be the most widely studied etiology [11, 12]. One case report described a patient with acute EBV infection with a positive direct Coombs test and a positive cold agglutinin test with identification shown to be specific for anti-I antibodies [6]. The I antigen is present in high titers in fetal red blood cells and is present in only small amounts in the erythrocytes of adolescents. Anti-I antibodies can agglutinate red blood cells at temperatures of up to 37°C if the titer level is sufficiently high. Although we were unable to perform a complete workup for hemolysis in this case, our patient followed the expected course with self-resolution of his symptoms within a few weeks [6, 13].

This case uniquely highlights gross hematuria and intravascular hemolysis as complications of infectious mononucleosis. It also suggests EBV infection should be considered in patients with pharyngitis and hematuria. Simple urine microscopy can help differentiate EBV-related hematuria from other conditions.

## 4. Results

The patient presented with significant gross hematuria, elevated white blood cell count, and elevated liver enzymes. Initial laboratory findings included a hemoglobin level of 15.4 g/dL, which decreased to 13.8 g/dL at a follow-up visit one week later. Urinalysis showed a high concentration of red blood cells and hemoglobinuria. Serological tests confirmed active EBV infection, and further tests indicated intravascular hemolysis, with elevated plasma hemoglobin and LDH levels. The patient's condition improved with supportive care, and his hematuria resolved within two weeks.

## Data Availability

The data that support the findings of this study are available from the corresponding author on request. The data are not publicly available due to privacy or ethical restrictions.

## References

[1] Lewis M. J. (1988). Gross hematuria as a manifestation of infectious mononucleosis. *Journal of Adolescent Health Care*.

[2] Lindsey D. C., Chrisman W. P. (1955). Gross hematuria as the presenting symptom of infectious mononucleosis. *JAMA*.

[3] Taub E. A. (1966). Renal lesions, gross hematuria, and marrow granulomas in infectious mononucleosis. *JAMA*.

[4] Brun C., Madsen S., Olsen S. (1970). Infectious mononucleosis with hepatic and renal involvement. *Scandinavian Journal of Gastroenterology-Supplement*.

[5] Fekete A. M., Kerpelman E. J. (1965). Acute Hemolytic anemia complicating infectious mononucleosis. *JAMA*.

[6] Thompson W. T., Pitt C. (1950). Frank hematuria as a manifestation of infectious mononucleosis. *Annals of Internal Medicine*.

[7] Tidy H. L., Morley E. B. (1921). Glandular fever. *BMJ*.

[8] Sumaya C. V., Ench Y. (1985). Epstein-barr virus infectious mononucleosis in children: I. Clinical and general laboratory findings. *Pediatrics*.

[9] Iwama H., Horikoshi S., Shirato I., Tomino Y. (1998). Epstein-Barr virus detection in kidney biopsy specimens correlates with glomerular mesangial injury. *American Journal of Kidney Diseases*.

[10] Madigan N. P., Newcomer A. D., Campbell D. C., Taswell H. F. (1973). Intense jaundice in infectious mononucleosis. *Mayo Clinic Proceedings*.

[11] Green N., Goldenberg H. (1960). Acute hemolytic anemia and hemoglobinuria complicating infectious mononucleosis. *Archives of Internal Medicine*.

[12] Einzig M. J., Neerhout R. C. (1969). Hemolytic anemia in infectious mononudeosis. *Clinical Pediatrics*.

[13] Chambers C. V., Irwin C. E. (1986). Intense jaundice in an adolescent: an unusual presentation of infectious mononucleosis. *Journal of Adolescent Health Care*.

